# Chest CT scan for the screening of air anomalies at risk of pulmonary barotrauma for the initial medical assessment of fitness to dive in a military population

**DOI:** 10.3389/fphys.2022.1005698

**Published:** 2022-10-07

**Authors:** Brieuc Bonnemaison, Olivier Castagna, Sébastien de Maistre, Jean-Éric Blatteau

**Affiliations:** ^1^ Service de Médecine Hyperbare et d’Expertise Plongée (SMHEP), Hôpital d'Instruction des Armées Sainte-Anne, Toulon, France; ^2^ Equipe de Recherche Subaquatique et Hyperbare, Institut de Recherche biomédicale des armées, Toulon, France; ^3^ Laboratoire Motricité Humaine Expertise Sport Santé, UPR 6312, Nice, France; ^4^ Cellule plongée humaine et Intervention sous la Mer (CEPHISMER), Force d’action navale, Toulon, France

**Keywords:** diving, pulmonary barotrauma, bullae, blebs, pulmonary cyst, CT chest, spirometry, decompression illness

## Abstract

**Introduction:** The presence of intra-pulmonary air lesions such as cysts, blebs and emphysema bullae, predisposes to pulmonary barotrauma during pressure variations, especially during underwater diving activities. These rare accidents can have dramatic consequences. Chest radiography has long been the baseline examination for the detection of respiratory pathologies in occupational medicine. It has been replaced since 2018 by the thoracic CT scan for military diving fitness in France. The objective of this work was to evaluate the prevalence of the pulmonary abnormalities of the thoracic CT scan, and to relate them to the characteristics of this population and the results of the spirometry.

**Methods:** 330 records of military diving candidates who underwent an initial assessment between October 2018 and March 2021 were analyzed, in a single-center retrospective analysis. The following data were collected: sex, age, BMI, history of respiratory pathologies and smoking, treatments, allergies, diving practice, results of spirometry, reports of thoracic CT scans, as well as fitness decision.

**Results:** The study included 307 candidates, mostly male, with a median age of 25 years. 19% of the subjects had abnormal spirometry. We identified 25% of divers with CT scan abnormalities. 76% of the abnormal scans were benign nodules, 26% of which measured 6 mm or more. Abnormalities with an aerial component accounted for 13% of the abnormal scans with six emphysema bullae, three bronchial dilatations and one cystic lesion. No association was found between the presence of nodules and the general characteristics of the population, whereas in six subjects emphysema bullae were found statistically associated with active smoking or abnormal spirometry results.

**Conclusion:** The systematic performance of thoracic CT scan in a young population free of pulmonary pathology revealed a majority of benign nodules. Abnormalities with an aerial component are much less frequent, but their presence generally leads to a decision of unfitness. These results argue in favor of a systematic screening of aeric pleuro-pulmonary lesions during the initial assessment for professional divers.

## 1 Introduction

Scuba diving is a practice with high constraints for the body, linked in particular to immersion in a cold, unbreathable environment and to the increase in ambient pressure. These conditions can be responsible for specific pathologies and accidents with, in particular, the possibility of barotraumatic pulmonary accidents which are particularly feared because of the serious complications which are often associated with them.

These barotraumatic accidents are related to the variations of the gas volume within the pulmonary alveoli as a function of the pressure, according to the Boyle-Mariotte law. When the airway pressure can no longer be balanced with that of the external environment, the risk is that of pulmonary overpressure causing alveolar lesions. The pressurized air then passes through the alveolar walls, diffuses into the surrounding spaces, i.e., the pleura and the mediastinum, and can enter the pulmonary circulation. Symptoms of pulmonary barotrauma (PBT) can be respiratory, neurological, and lead to death (Neuman et al., 1998; Lippmann et al., 2011; Bralow and Piehl 2018).

Epidemiologically, pulmonary barotrauma accidents are uncommon, representing 5% of diving accidents admitted to hyperbaric centers (Coulange et al., 2008). Nevertheless, severe forms with cerebral aero-embolism are observed in 18% of cases, sometimes with life-threatening consequences (Neuman et al., 1998; Lippmann et al., 2011). The majority of cases occur during effort dives, particularly in the context of an ascent with expiratory blockage (Coulange et al., 2008; Lafère et al., 2009).

Although PBT most often occur in the absence of any pre-existing lung pathology, the presence of certain anomalies with an air component such as pulmonary cysts, blebs (sub-centimeter collections of air) or emphysema bubbles predisposes to this type of accident (Reuter et al., 1997; Tetzlaff et al., 1997). The proportion of air formations resulting in pulmonary overpressure in the general population is not known. A retrospective study conducted within fifteen cases of PBT that occurred in the military diving school found scannographic air formations in two cases (13.3%). These PBT episodes were due in 73% of the cases to an exhalation ascent exercise, justifying the change in the way this exercise was conducted in 2017.

The military has approximately 2,200 professional divers. The prevention of military diving accidents is based on enhanced medical surveillance with initial and periodic medical examinations. During the initial medical examination of military divers, various elements that may contraindicate the practice of professional diving are sought. The spirometry allows the detection of obstructive ventilatory disorders (COPD, asthma). Since October 2018, the prescription of a low-dose chest CT scan during the initial visit replaces the chest X-ray previously performed annually. Chest CT scan indeed has good sensitivity (between 84% and 88%) and specificity (close to 100%) to detect emphysema blebs and bullae in the setting of primary spontaneous pneumothorax (PSP) (Hatz et al., 2000; Sihoe et al., 2000; Chou et al., 2003; Margolis et al., 2003; Kawaguchi et al., 2013; Almajid et al., 2019) or COPD (Newell 2002). It is more efficient than chest radiography in detecting these abnormalities (Sihoe et al., 2000; Newell 2002; Coulange et al., 2008). In addition, low-dose lung CT is an inexpensive and less radiative examination than the usual CT scan, for equivalent performance in detecting lung lesions (Corneloup et al., 2003; Ferretti and Jankowski. 2010). Numerous studies have evaluated the proportion of preexisting lung abnormalities in subjects with PSP (Smit et al., 2000; Haynes and Baumann 2011; Grundy et al., 2012; Casali et al., 2013; Casha et al., 2014; Lyra 2016), mainly bullae and emphysema blebs. However, there are few data on the presence of intrapulmonary air anomalies in the general population (Smit et al., 2000).

The primary objective of this study was to quantify lung abnormalities on screening chest CT in a population of military diver candidates free of known lung pathology. It also aimed to determine the prevalence of pulmonary air formations that might contraindicate scuba diving.

## 2 Materials and methods

### 2.1 Study population

**FIGURE 1:**
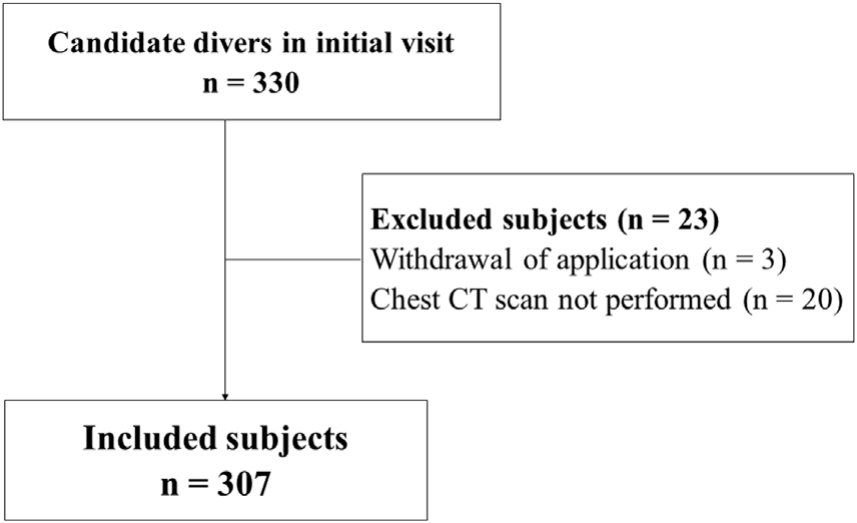
Flow chart.

This was a retrospective study. Data were those of military professional diving candidates whose initial visit was performed between October 2018 and March 2021. Records of candidates who did not receive a chest CT scan, as well as those who withdrew their application, were excluded. Thus, 23 subjects were excluded from the study among the 330 who were admitted at the initial visit (Figure 1). The files were all from the hyperbaric medicine and diving expertise service of the Sainte-Anne military hospital, Toulon, the referent center for the expertise of military divers in France.

### 2.2 Data collection

The data collected from the medical records were: sex, age, weight, height, BMI, the presence of a history of respiratory diseases, the history of smoking (weaned or not), the taking of treatments, the presence of allergies, the practice of diving, regular or anecdotal, the results of the spirometry (using MIR Spirolab III) and more precisely the ratio of the measured FEV1 (forced expiratory volume in one second) to the theoretical FEV1 (in %), the measured midexpiratory flow at 50% (MEF50) to the theoretical MEF50 (in %) and the relation of measured FEV1 in percent of measured forced vital capacity (FVC). Values were considered abnormal for a measured FEV1/theoretical FEV1 less than 0.9, a measured MEF50/theoretical MEF50 less than 0.75, and a FEV1/FVC less than 0.75. A pathological CT scan result was also sought with, if necessary, the analysis of the lesions observed. Finally, the final decision of fitness to dive was noted.

### 2.3 Imaging techniques

Images were obtained using a low-dose GE Revolution CT multi-slice scanner (slice thickness 0, 625/mm). Pathological findings were analyzed in terms of emphysematous, cystic, bronchial (bronchial dilatation, bronchial syndrome, bronchial thickening) and parenchymal (nodules with distinction concerning nodules larger than 6 mm, granulomas, atelectasis, ground glass abnormalities, ventilatory disorders) abnormalities. The radiologists working in the hospital are trained in imaging related to diving medicine.

### 2.4 Statistical analysis

The main analysis consisted of identifying the different imaging abnormalities and establishing their prevalence. A secondary analysis looked for an association between an abnormal imaging finding, regardless of the abnormality, and the spirometry findings. Two additional analyses were performed to look for possible relationships between individual subject characteristics or spirometry data and the presence of chest CT scan nodules or emphysematous lesions. The distribution of the variables being heterogeneous and not necessarily following a normal distribution, non-parametric tests were preferred. Data are expressed as medians and interquartile ranges (Q1–Q3) or as frequencies (%). The Mann-Whitney test was used to compare continuous variables. The chi-square test was used for qualitative variables. A *p* value <0.05 was considered significant. Statistical analyses were performed using the GraphPad Prism 9 application.

## 3 Results

### 3.1 Description of the population

Table 1 describes the characteristics of the study population. The vast majority were men (96.1%), with a median age of 25 years. Few had a history of respiratory disease (6.5%), allergies (17%), or medication use (2%). 22.5% of subjects had a history of smoking and 10.5% were active smokers. A total of 58 abnormal spirometry results were identified, of which only three resulted in a decision of unfitness. The median FEV1/FVC ratio was 83.3% (78.8–88.7), median FEV1 103% of theoretical value (98–109) and MEF50 94% of theoretical value (81.2–109).

**TABLE 1:** Characteristics of the study population.

	
Gender	
Male (n, %)	295 (96.1%)
Female (n, %)	12 (3.9%)
Age (median, Q1–Q3)	25 years (23–28)
BMI (median, Q1–Q3)	23.8 kg m^−2^ (22.2–25.1)
Respiratory history (n, %)	20 (6.5%)
Smoking history (n, %)	69 (22.5%)
Unweaned smoking (n, %)	32 (10.5%)
Taking of treatments (n, %)	6 (2%)
Allergies (n, %)	52 (17%)
Practice of diving (n, %)	207 (67.4%)
Spirometry results	
FEV1/VC (median, Q1–Q3)	83.3% (78.8–88.7)
Measured FEV1/theoretical FEV1 (median, Q1–Q3)	103% (98–109)
Measured MEF50/theoretical MEF50 (median, Q1–Q3)	94% (81.2–109)

### 3.2 Imaging results

**FIGURE 2:**
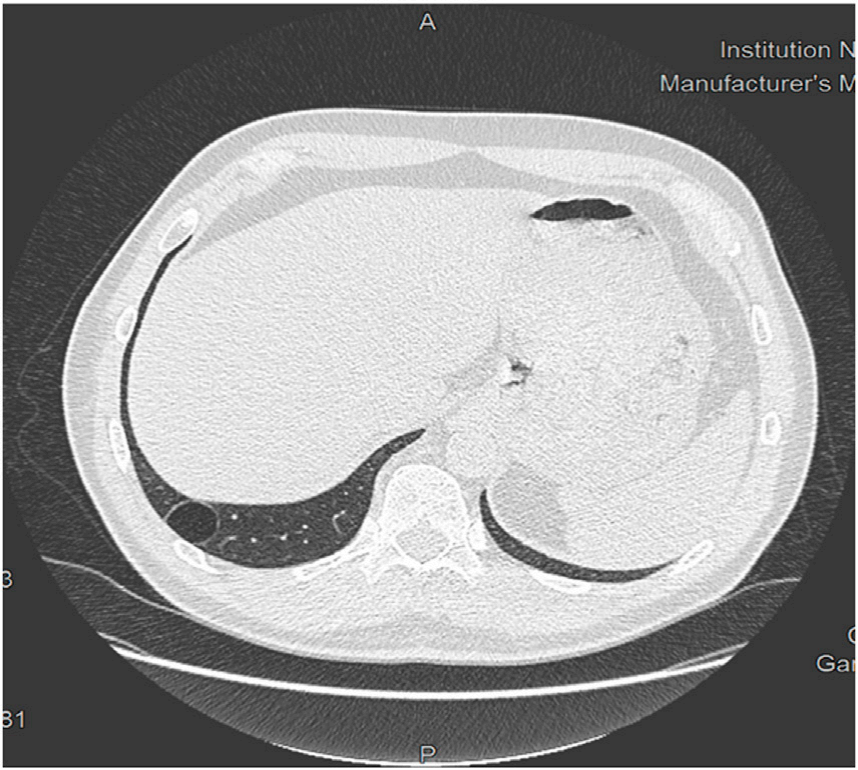
Chest CT scan showing a right basal subpleural cyst.

**FIGURE 3:**
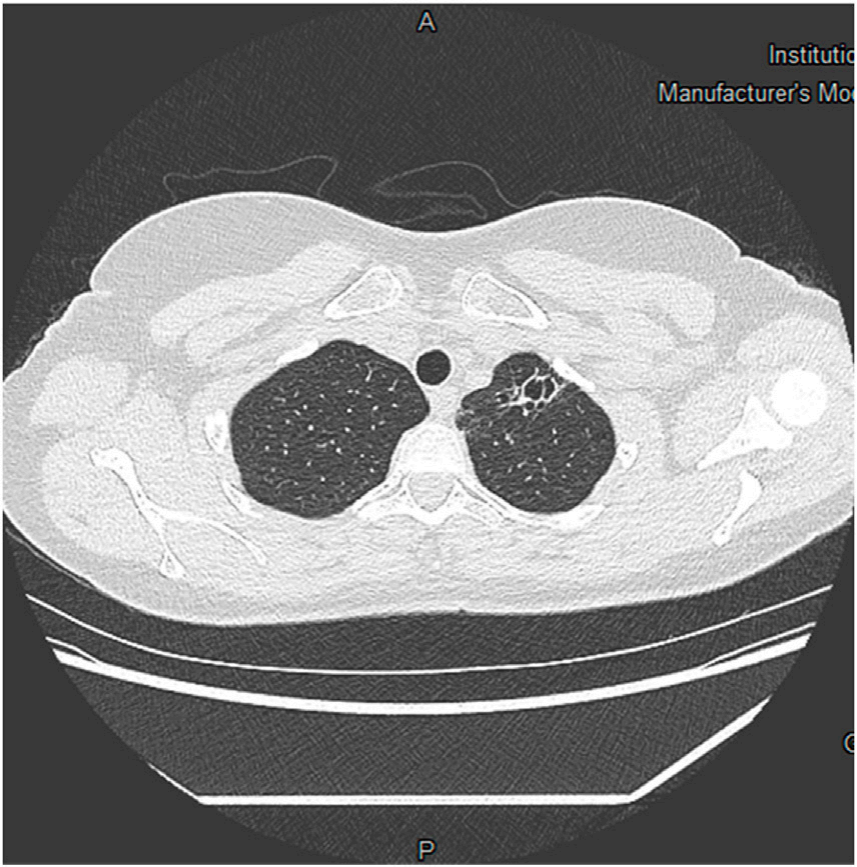
Chest CT scan showing apical bronchiectasis.

76 chest CT scans (24.8%) had a total of 86 abnormalities, presented in Table 2. These abnormalities were largely dominated by nodules (*n* = 58, 76.3% of abnormal CT scans), all of which were solid in consistency and benign in appearance. Of these, 15 nodules (25.9%) were 6 mm in long axis or more. Six CT scans showed emphysematous lesions, exclusively bullae. Four involved apical peripheral regions, whereas two were perihilar or laterobasal. Only one was unique. All were infracentimetric. Only one CT scan with an emphysema image did not result in an unfitness decision. A subpleural cyst was found in one CT scan (Figure 2), and resulted in a decision of unfitness. Bronchial dilatations were diagnosed in three CT scans (Figure 3), and resulted in a decision of unfitness for these three cases.

**TABLE 2:** Prevalence of abnormalities found on chest CT scan.

	Number of anomalies	% of CT scans anomalies (*n* = 76)	% of anomalies in the study population (*n* = 307)
Emphysema	6	7.9	2.0
Cysts	1	1,3	0.3
Bronchial anomalies			
Bronchial dilatation	3	3.9	1.0
Bronchial syndrome	2	2.6	0.7
Bronchial thickening	2	2.6	0.7
Parenchymal abnormalities			
Nodules (including ≥ 6 mm)	58 (15)	76.3	18.9
Granulomas	7	9.2	2.3
Atelectasis	2	2.6	0.7
Ground glass	2	2.6	0.7
Ventilatory disorders	2	2.6	0.7
Irregularity of the apices	1	1.3	0.3
Total	86		28

### 3.3 Secondary analysis

#### 3.3.1 Comparison between an abnormal CT scan result with different features and the results of the spirometry

The results of the comparison between an abnormal CT scan result, regardless of the abnormality, and either population characteristics or an abnormal spirometry result are transcribed in Table 3. There was no significant association.

**TABLE 3:** Characteristics of subjects according to a normal or abnormal CT scan result.

	Normal CT scan	Abnormal CT scan	*p-*value
Gender			> 0.99
Male (n, %)	222 (96.1%)	73 (96.1%)	
Female (n, %)	9 (3.9%)	3 (3.9%)	
Age (median, Q1–Q3)	25 (23–28)	25 (22.5–28)	0.81
BMI (median, Q1–Q3)	23.7 (22, 3–25)	24 (22–25)	0.57
Respiratory history (n, %)	15 (6.5%)	3 (4%)	0.43
Smoking history (n, %)	49 (21.2%)	20 (26.7%)	0.33
Unweaned smoking (n, %)	22 (9.5%)	10 (13.3%)	0.35
Taking of treatments (n, %)	5 (2.2%)	1 (1.3%)	>0.99
Allergies (n, %)	42 (18.2%)	10 (13.3%)	0.43
Practice of diving (n, %)	152 (65.8%)	54 (0.7%)	0.32
Abnormal spirometry (n, %)	39 (17%)	19 (25.3%)	0.10
FEV1/FVC (median, Q1–Q3)	83.6 (80–89)	82 (77–88.3)	0.10
Measured FEV1/theoretical FEV1 (median, Q1–Q3)	103 (98–109)	102 (95–110)	0.37
Measured MEF50/theoretical MEF50 (median, Q1–Q3)	94 (82–109)	95 (78–109)	0.50

#### 3.3.2 Nodule analysis

There was no significant association between the presence of nodules and an abnormal spirometry result (*p* = 0.46), nor between the presence of nodules and smoking (*p* = 0.23). No associations were found either by dissociating the different spirometry values or by considering separately nodules measuring 6 mm of long axis or more.

#### 3.3.3 Emphysema bullae analysis

**FIGURE 4:**
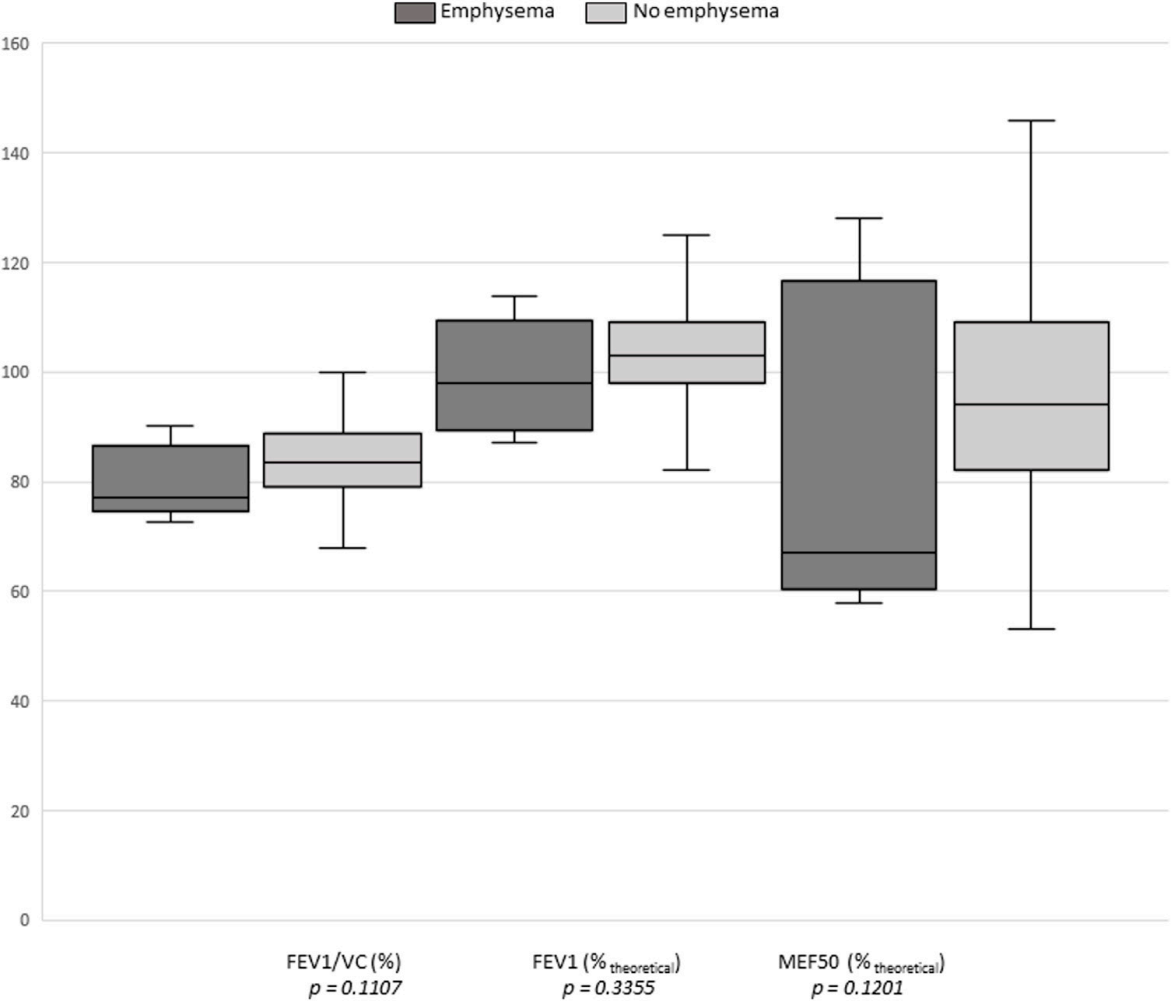
Graphical representation of the comparison between a CT scan emphysema result and the three spirometry criteria.

The presence of emphysema on CT scan was significantly associated with active smoking (OR = 9.3; *p* = 0.0014) (Table 4), as well as with an abnormal spirometry result (OR = 9.1; *p* = 0.0026). Separate analysis of the three spirometry parameters showed no significant associations (Figure 4).

**TABLE 4:** Characteristics of subjects according to the presence of CT scan emphysema.

	No emphysema	Emphysema	Odds ratio (95% CI)	*p*-value
Smoking history (n, %)	66 (22%)	3 (50%)		0.10
Unweaned smoking (n, %)	29 (9.7%)	3 (50%)	9,3 (2.1–41)	**0.0014**
Abnormal spirometry (n, %)	54 (18%)	4 (66.7%)	9.1 (2.1–48.4)	**0.0026**
FEV1/FVC (median, Q1–Q3)	83.4 (79–88.8)	77 (75.2–83.7)		0.11
Measured FEV1/theoretical FEV1 (median, Q1–Q3)	103 (98–109)	98 (91–106.5)		0.33
Measured MEF50/theoretical MEF50 (median, Q1–Q3)	94 (82–109)	67 (62–113)		0.12

Bold values mean *p* < 0.05.

Gender did not appear to have a significant association with the presence of an abnormal chest CT (all abnormalities combined; *p* > 0.99).

#### 3.3.4 Unfitness to dive

Of the 306 subjects included, 20 were declared unfit for military scuba diving:

- five of the six subjects with emphysematous lesions were declared unfit because of the potential risk of pulmonary barotrauma;
- one subject because of bronchial dilatations also at risk;
- one subject had a pulmonary vascular malformation on the CT scan;
- one subject had a history of significant asthmatic bronchitis;
- three subjects had obstructive ventilatory disorders on the spirometry;
- four subjects were disqualified for ENT reasons: two for hearing loss and two for tubal dyspermeability with failure of the functional recompression test;
- one subject had a disc protrusion considered to be at risk of spinal cord decompression sickness;
- two subjects were declared unfit because of a significant decrease in visual acuity;
- one subject had a history of exercise-related heat illness;
- one subject had a history of psychiatric illness.

## 4 Discussion

### 4.1 Nodules issue

In our study, scannographic abnormalities were overwhelmingly dominated by intra-pulmonary nodules. One or more nodules were present in 76% of abnormal CT scans, representing 19% of the study population. Although the presence of nodules is not associated with an increased risk of PBT in diving, it is important to identify criteria for malignancy and surveillance that could have medical implications for professional diving.

#### 4.1.1 Prevalence of incidental nodules in the general population

Several reviews of the literature from the United States report highly variable prevalences of nodules at CT scans screenings, ranging from 3% to 51% depending on the study, with an overall average of approximately 20% (Wahidi et al., 2007; Bach et al., 2012). The steady increase in detection of these abnormalities may be explained by both easier access to chest CT scan and their better sensitivity (Gould et al., 2015).

#### 4.1.2 Relationship between spirometry and presence of pulmonary nodules

Consistent with the literature, our study found no relationship between the presence of pulmonary nodules and subject characteristics, including smoking history (*p* = 0.17). There was also no significant relationship between the presence of nodules and an abnormal spirometry (*p* = 0.46), whether it was FEV1/VC, FEV1 or MEF50 (*p* = 0.24, *p* = 0.41, *p* = 0.48, respectively).

#### 4.1.3 Management after detection of nodules

There does not appear to be an association in the cited literature between smoking and the occurrence of nodules, either in terms of prevalence or size (Bach et al., 2012; Gould et al., 2015; Boldeanu et al., 2018). Nevertheless, according to the 2017 Fleischner Society recommendations, heavy smoking is among the criteria that will make one discuss radiological follow-up of the nodule(s) as a risk factor for lung cancer (MacMahon et al., 2017). The Fleischner Society recommendations apply only to a population with an age greater than or equal to 35 years and advocate distinguishing solid nodules from those with ground-glass or solid components, before analyzing size and number. The management algorithm differs according to the level of risk of malignancy of the nodule(s). Size and morphology are strongly related to the risk of malignancy (Wahidi et al., 2007; Austin 2011). In our study, the majority of nodules were less than 3 mm, benign in appearance. Only 26% of scans had one or more nodules 6 mm or larger. Smoking was not statistically related to the presence of nodules (*p* = 0.17). Of the pathologic CT scans that were secondarily controlled, no suspicious cases were detected.

### 4.2 Emphysema bullae issue

#### 4.2.1 Prevalence of emphysema lesions in primary spontaneous pneumothorax episodes

There is a significant association between the presence of pleural abnormalities and the occurrence of PSP. These abnormalities, mainly subpleural bullae and blebs, are reportedly present on CT scans in 56% of PSP cases and in 60.8%–68.1% of recurrences, with an incidence proportional to the degree of dystrophic involvement (Smit et al., 2000; Casali et al., 2013; Riveiro-Blanco et al., 2020). Other studies describe a prevalence of emphysema between 76% and 100% in thoracoscopies performed after an episode of PSP (Grundy et al., 2012; Casha et al., 2014). Pleural porosity lesions have also been described, where an inflammatory fibroelastic layer replaces the mesothelial cells of the visceral pleura, promoting the passage of air from the alveolus to the pleura (Haynes and Baumann 2011).

#### 4.2.2 Prevalence of incident emphysema lesions in the general population

In the general population, emphysematous lesions are the second most common incidental pulmonary finding, behind non-calcified nodules. The observed prevalences vary from 11% to 16% (Priola et al., 2013; Hussien et al., 2016; Boldeanu et al., 2018) and even up to 50.6% (Morgan et al., 2017). However, these studies included subjects in their 70s, most with major smoking at more than twenty pack-years. One study identified a 6% prevalence of bullae or blebs in thoracotomy surgeries performed in patients aged 15–51 years without pulmonary pathology, a prevalence that may be underestimated (Amjadi et al., 2007). Smoking appears to play a major role in the formation of emphysematous lesions, a fortiori in combination with other toxic substances such as cannabis or cocaine (Almeida et al., 2015; Underner et al., 2018). A Dutch postmortem study of 130 adults aged 21–70 years and all causes of death found a 34% CT scans prevalence of emphysema blebs and bullae, increasing with age (de Bakker et al., 2020). A study conducted at the Royal Netherlands Navy Diving Medical Center compared the sensitivity of chest CT scan to chest radiography in asymptomatic male military subjects (Wingelaar et al., 2021). The characteristics of the study population were similar to those of our study, with subjects of mean age 36.4 years, compared with 25 years in our study. Among the 101 scans performed, seven incidentalomas (7%) with blebs or emphysema bullae were detected, compared with 2% in our study. Five of the seven subjects with emphysematous lesions were declared unfit for work, which is approximately the same proportion as in our study. In this study, CT scan was significantly more effective than chest radiography in detecting emphysematous lesions in asymptomatic subjects (*p* = 0.023). A South Korean study of 536 male civilian pilot candidates aged 45–51 years described similar results (Bang et al., 2012). Routine screening with low-dose chest CT scans yielded a 6.1% prevalence of aerial formations, including 5.8% of emphysematous blebs or bullae lesions.

#### 4.2.3 Relationship between spirometry and presence of emphysema

Emphysema lesions result from inflammation and destruction of the lung parenchyma (Egger and Aubert, 2005). Parenchymal destruction amputates the exchange surface between alveolar capillary blood and alveolar gas. This altered alveolar-capillary exchange, which is prognostically critical in emphysema, is expressed in spirometry as decreased carbon monoxide transfer (DLCO) (Bates 2000; Bodlet et al., 2013; Cottin 2013; Amariei et al., 2019). The obstructive ventilatory abnormality results from the rarefaction of parenchymal fibers that support the bronchial walls, which promotes their collapse especially during expiratory efforts. Several studies highlight the deterioration of FEV1 and FEV1/VC ratio due to emphysema (Bodlet et al., 2013; Amariei et al., 2019), an association that is more marked the more severe the impairment (Sanders et al., 1988; Kitaguchi et al., 2014; Crossley et al., 2018). For the detection of emphysematous lesions, the sensitivity of spirometry is much lower than that of the chest CT scan (Sanders et al., 1988; Madani et al., 2001). In a study of subjects with PBT, five out of fifteen (33%) had subpleural emphysema bullae (Tetzlaff et al., 1997). In the same study, peak expiratory flow rates at 25 and 50% of vital capacity were lower in subjects with PBT than in those without (*p* < 0.05 and *p* < 0.02, respectively). Nevertheless, the small number of subjects included in this study did not allow generalization of these results. Spirometry abnormalities may vary not only with the degree of involvement, but also with the type of involvement. FEV1 and residual volume seem to be preferentially affected in emphysematous lesions of the lower lobes, whereas DLCO would be more affected if the lesions involve the upper lobes (Haraguchi et al., 1998). In addition, central lung involvement is more likely to result in altered spirometry, compared to peripheral involvement (Nakano et al., 1999). In our study, the presence of emphysema lesions was significantly associated with alterations in spirometry (OR = 9.1; *p* = 0.0026). There was no significant association between the presence of emphysema and FEV1/FVC ratio (*p* = 0.11), FEV1 (*p* = 0.34), or MEF50 (*p* = 0.12). In our study, of the six emphysematous lesions, four were apical, two peri-hilar and latero-basal. They were all minimal. This low severity and their location could explain the absence of significant alteration of the spirometry variables analyzed. However, the small number of subjects (six with emphysema bullae) does not allow us to draw any conclusion on the spirometric abnormalities observed.

### 4.3 Other pulmonary air anomalies

#### 4.3.1 Prevalence of pulmonary cystic lesions

Pulmonary cysts are defined as round, usually thin-walled, intraparenchymal lesions with a well-defined interface with the healthy lung. They are also a source of PBT, and may be a consequence of lung aging. The existence of these lung cysts can have serious consequences (reference Dufresne et al., 2020). A study in the United States of America of a large cohort of 2,633 asymptomatic subjects aged 34–92 years estimated the prevalence at 7.6% (Araki et al., 2015). Age plays an important role. Indeed, the mean age of subjects with at least one cyst was 63 years versus 58.9 years in subjects without cysts (*p* < 0.001), and no cysts were found in subjects younger than 40 years. While the prevalence of cystic lesions in the general population is poorly described in the literature, it is significantly more so for special cases (Gupta et al., 2013; Gupta et al., 2015; Johannesma et al., 2016). Some genetic syndromes predispose to it, such as Birtt-Hogg-Dubé syndrome, in which these lesions are reportedly present in more than 80% of subjects, or neurofibromatosis, langerhansian histiocytosis, and lymphangioleiomyomatosis. Gougerot-Sjögren’s syndrome is associated with the presence of cysts in 12%–46% of cases.

#### 4.3.2 Prevalence of bronchial dilatation

A South Korean study observed a prevalence of asymptomatic bronchiectasis at 2,300 cases per 100,000 (Kim et al., 2021). Their presence appeared to be statistically related to female gender, older age, liver comorbidity, a history of tuberculosis or COPD, or decreased FEV1. In a UK study, the prevalence was lower but increased over time, particularly in older age groups (Quint et al., 2016). Histological abnormalities of bronchial dilatation are consistent with chronic inflammatory disease with impaired clearance facilitating pulmonary superinfections and chronic inflammation. The severity of these bronchiectasis increases with advancing age. Other risk factors for severity include decreased FEV1, low BMI, recent hospitalization or cognitive impairment, and ischemic heart disease (Quinn and Hill 2018). PBTs in relation to bronchiectasis are poorly described, but airway abnormalities are a histologic weakness during pressure changes.

### 4.4 Screening chest CT scan

#### 4.4.1 Performance of low- and ultra-low-dose CT to detect bullous lesions

High-resolution CT scans are effective in detecting emphysema bullae and blebs, with sensitivity compared with surgery ranging from 84% to 96% (Sihoe et al., 2000; Kawaguchi et al., 2013; Almajid et al., 2019). Regarding low-dose CT, it is already commonly used for screening of smoking subjects at risk for lung cancer. In these elderly, smoking cohorts, the prevalences are 44%–50% for emphysematous lesions, and 14% for bronchiectasis (Morgan et al., 2017; Regan et al., 2019). The prevalence of emphysematous lesions in COPD patients followed by low-dose CT was 27% in the study by Smith et al. (2014) and 78% in the study by Alcaide et al. (2017) in a general smoking population. For emphysema screening, the diagnostic qualities of low-dose CT appear to be equivalent to conventional CT with a difference in sensitivity of less than 3% (Zaporozhan et al., 2006; Gierada et al., 2007; Ohno et al., 2016). Over the past 5 years, studies of “ultra-low dose” CT, which has a radiation level between that of a chest X-ray and low-dose CT, have increased (Messerli et al., 2017; Svahn et al., 2019). The emphysema quantification capabilities of ultra-low-dose CT appear to match those of a standard CT scan, despite possible slight underestimation, with 84% less radiation (Messerli et al., 2017; Wisselink et al., 2021). Comparison of the results of low-dose scans with those of ultra-low-dose scans appears to demonstrate similar performance in detecting lung abnormalities such as nodules, bronchiectasis, and emphysematous lesions, with the latter having a sensitivity greater than 65% (Kim et al., 2015; Wang et al., 2015; Tækker et al., 2021).

#### 4.4.2 Impact of these imaging tests in terms of radiation and finances

For comparable diagnostic results, the average radiation for a standard chest CT is 9.0 mSv, compared with 1.8 mSv for a low-dose CT (Leitão et al., 2021). Ultra-low-dose chest CT would have mean irradiations between 0.07 and 0.22 mSv, which is between those of a standard X-ray and a low-dose CT (Mettler et al., 2008; Larke et al., 2011; Kim et al., 2015; Wang et al., 2015; Messerli et al., 2017; Rampinelli et al., 2017; Kroft et al., 2019; Azadbakht et al., 2021; Leitão et al., 2021). In financial terms, the cost of thoracic CT (standard or low-dose) is slightly higher than that of thoracic radiography: €25.27 versus €21.82 for fee-for-service plus the technical package, ranging from €32 to €93.03 (in France) depending on the type of device and its activity.

### 4.5 Strengths and limitations of the study

This is one of the first retrospective studies to examine the prevalence of pulmonary incidentalomas and their potential association with spirometry findings in a young, healthy population. The aerial abnormalities detected by CT examination, including emphysema lesions at risk of PBT, would likely not have been detected on chest radiography. In this young, healthy population, there are few confounding factors. This study has some limitations. First, because it is a retrospective study, the level of evidence that can be expected is lower than that of a prospective study. Second, the study population is very selective, made up of young, athletic, healthy military subjects. Indeed, military diver candidates are not representative of the general population of professional or recreational civilian divers. A study in a less narrowly selected population would likely strengthen the associations found, particularly between scannographic abnormalities and spirometry. It might also be interesting to compare it with a control population, for example, military subjects of the same age who do not practice diving. In addition, the narrow age range does not allow observation of the longer-term consequences of smoking on the lung parenchyma. Concerning the spirometry standards, we used the European Coal and Steel Community (CECA) standards, because these are the values still used for fitness to dive assessment in the French army. They have a good reliability compared to the GLI-2012 values in a population of 18–40 years old. However, the spirometry values seem to be underestimated in a population >40 years old, especially women. However, our study population has a median age at 25 years, with only one male subject >40 years (42 years). Perhaps the additional measurement of DLCO could raise the sensibility for the detection of asymptomatic emphysema. Finally, a larger-scale study would provide additional power.

## 5 Conclusion

The systematic performance of a thoracic CT scan for the fitness of military professional diving candidates revealed the existence of nodular lesions, all benign and without significant association with the characteristics of the study population. A second type of less frequent abnormalities consisted of aerial formations such as cysts, emphysema bubbles, and bronchial dilatations. In a subgroup analysis, a significant association was observed between the presence of emphysema and unweaned smoking, as well as with an abnormal spirometry result. However, these results should be considered in light of the limited sample size and the lack of current confirmation in the literature. In addition, there is no description in the literature of an association for other air anomalies potentially at risk for PBT such as pulmonary cysts or bronchiectasis. The non-negligible prevalence of these abnormalities in the general population, especially over 40 years of age, and the absence of clinical signs, argue in favor of performing a chest CT scan.

Given the increasing development of low-dose CT and the results presented in this work, the systematic performance of an initial thoracic CT scan in civilian professional divers, who may be required to make ascents in emergency situations, seems relevant. In the field of recreational diving, while waiting for the emergence of ultra-low dose scanners, the performance of a thoracic CT scan could be considered according to age and/or in the case of points of call, such as the presence of respiratory symptoms or history, unweaned or significant smoking, or abnormal spirometry results. The addition of DLCO measurement could be contributory in some cases, but would require greater accessibility to this measurement. Thus, detection of lung injury now appears to rely on the widespread use of CT imaging. The progressive access to ultra-low dose lung scanners should further facilitate this use.

## Data Availability

The raw data supporting the conclusion of this article will be made available by the authors, without undue reservation.
